# The Effect of a Simulated Commercial Flight Environment with Hypoxia and Low Humidity on Clotting, Platelet, and Endothelial Function in Participants with Type 2 Diabetes – A Cross-over Study

**DOI:** 10.3389/fendo.2018.00026

**Published:** 2018-02-13

**Authors:** Judit Konya, Benjamin E. J. Spurgeon, Ahmed Al Qaissi, Thozhukat Sathyapalan, Ramzi Ajjan, Leigh Madden, Khalid M. Naseem, Andrew Thomas Garrett, Eric Kilpatrick, Stephen L. Atkin

**Affiliations:** ^1^Department of Academic Endocrinology, Diabetes and Metabolism, Hull York Medical School, Hull, United Kingdom; ^2^School of Medicine, Leeds Institute for Cardiovascular and Metabolic Medicine, University of Leeds, Leeds, United Kingdom; ^3^Hull York Medical School, University of Hull, Hull, United Kingdom; ^4^Department of Sport, Health and Exercise Science, University of Hull, Hull, United Kingdom; ^5^Sidra Medical Research Centre, Doha, Qatar; ^6^Department of Medicine, Weill Cornell Medicine Qatar, Doha, Qatar

**Keywords:** type 2 diabetes, hypoxia, flight simulation, platelet function, endothelial function, clotting

## Abstract

**Aims:**

To determine if clotting, platelet, and endothelial function were affected by simulated short-haul commercial air flight conditions (SF) in participants with type 2 diabetes (T2DM) compared to controls.

**Methods:**

10 participants with T2DM (7 females, 3 males) and 10 controls (3 females, 7 males) completed the study. Participants were randomized to either spend 2 h in an environmental chamber at sea level conditions (temperature: 23°C, oxygen concentration 21%, humidity 45%), or subject to a simulated 2-h simulated flight (SF: temperature: 23°C, oxygen concentration 15%, humidity 15%), and crossed over 7 days later. Main outcome measures: clot formation and clot lysis parameters, functional platelet activation markers, and endothelial function measured by reactive hyperemia index (RHI) by EndoPAT and serum microparticles.

**Results:**

Comparing baseline with SF conditions, clot maximal absorption was increased in controls (0.375 ± 0.05 vs. 0.39 ± 0.05, *p* < 0.05) and participants with T2DM (0.378 ± 0.089 vs. 0.397 ± 0.089, *p* < 0.01), while increased basal platelet activation for both fibrinogen binding and P-selectin expression (*p* < 0.05) was seen in participants with T2DM. Parameters of clot formation and clot lysis, stimulated platelet function (stimulated platelet response to ADP and sensitivity to prostacyclin), and endothelial function were unchanged.

**Conclusion:**

While SF resulted in the potential of denser clot formation with enhanced basal platelet activation in T2DM, the dynamic clotting, platelet, and endothelial markers were not affected, suggesting that short-haul commercial flying adds no additional hazard for venous thromboembolism for participants with T2DM compared to controls.

## Introduction

The environmental conditions on an aircraft result in reduced barometric pressure with a concomitant decrease in the partial pressure of oxygen resulting in lowering of the gas exchange in the lungs causing hypoxia. Aircraft cabins are pressurized to maintain good oxygenation, and the cabin altitude is around 8,000 ft (2,438 m). The humidity is kept low at 10–20% compared to 40–50% seen in an average building at sea level (1). The conditions inside an aircraft are irrespective of the flying altitude. Air travel was shown to increase the risk to develop venous thromboembolism (VTE) when risk factors are present (2), and traveler’s thrombosis is defined as a VTE occurring within 4 weeks after long-haul travel or occurring during the travel: the term “air-travel thrombosis” is used when the main part of the travel is air travel (3). Passengers have a medium risk to develop traveler’s DVT if they have two or more risk factors that include pregnancy or post-partum period, age over 60 years, documented thrombophilia, a family history of VTE, large varicose veins and/or chronic venous insufficiency, use of the oral contraceptive pill (OCP) or hormone replacement therapy (HRT), body mass index over 30. Thrombophilia or OCP use increases the risk to 14- to 16-fold (4). The presence of risk factors such as previous VTE, manifest malignant disease or other severe illness, immobilization or recent major surgery represents a high risk to develop traveler’s DVT. In passengers without the above risk factors, a prolonged journey slightly but indeterminately increases the risk of VTE (3). There is no evidence suggesting an association between hydration status and the development of VTE (5).

Evidence suggests that not only air travel, but any long-distance travel increases the risk of VTE development (6); however, when additional risk factors are present, the risk is increased with air travel (7). A case–control study estimated the risk of fatal pulmonary embolism following a flight of at least 3 h’ duration was between 0.5 and 0.6 per million passengers (8). In a hypobaric hypoxia study involving 20 healthy male volunteers, the authors showed that prothrombin 1 and 2 fragment levels reached a maximum level at 2 h into exposure (9). The evidence that participants with diabetes may be more likely to develop a VTE than healthy individuals is supported in some studies (10, 11), but not others (12). The development of a VTE may be contributed to in type 2 diabetes (T2DM) through a more compact clot structure through a smaller pore size and increased fiber thickness with more branch points (13), compared to on diabetes patients.

During blood clot formation, one of the most important steps is platelet activation that is known to be impaired in participants with T2DM. Platelet function abnormalities such as hypersensitivity of platelets to aggregant factors and hyposensitivity to anti-aggregants are present in participants with T2DM contributing toward increased platelet activity at locations of endothelial damage (14, 15).

Hypoxia can affect the endothelium and blood clot formation that can increase the risk for thrombosis. Endothelial microparticles (EMPs) are vesicles shed by the endothelial cells (EC) and consist of cell membrane, cytoplasmic, and nuclear elements. EMPs express surface markers reflecting their cell of origin (16). Under normal physiological conditions microparticles (MPs) are constantly shed into the circulation of healthy individuals (17). However, the level of EMPs is elevated in the circulation after damage, activation, or apoptosis of the EC, such as that seen for acute coronary syndromes and diabetes (18), and *in vivo* correlate with T2DM complications (19).

Short haul travel has not been investigated and specific studies exploring the risk for patients with diabetes to develop travel-related VTE are lacking. Given that hypobaric hypoxia showed prothrombotic changes at 2 h into exposure (9), this pilot study aimed to determine whether a simulated flight (SF) of 2 h duration in a hypoxic environment caused impairment in clotting indices, platelet function, and endothelial function, and whether this impairment was greater in participants with T2DM compared to healthy participants.

## Materials and Methods

All participants signed an informed consent form prior to their inclusion in the study.

Patients with T2DM were eligible for participation if they had a diagnosis of T2DM based on the WHO guidelines, they were on stable medication for diabetes during and for 3 months prior to the study (metformin being the only treatment permitted), age between 45 and 75 years at the beginning of the study, women postmenopausal and not on HRT, no hypo- hyperglycemia requiring medical attention or diabetes-related hospitalization in the preceding 12 months. Healthy volunteers were eligible for participation if aged 45–75 at the start of the study and women being postmenopausal and not on HRT. Exclusion criteria for both groups included the presence of any medical condition or concurrent medication that would interfere with the results of the study (i.e., antiplatelet medication or anticoagulants, previous VTE), any major clinical event in the preceding 3 months, participants not wishing to disclose their participation to their registered General Practitioner, HbA1c > 9%, smokers, claustrophobia, or panic attacks, subjects traveling by airplane or prolonged travel during the study or in the previous 4 weeks.

Twenty seven participants were screened for this study. Six participants failed screening or withdrew from the study (medication that affect platelet function [cyclooxygenase-2 inhibitor, *n* = 1; aspirin, *n* = 1], acute phase reaction at screening [*n* = 1], angina [*n* = 1], chronic obstructive pulmonary disease [*n* = 1] time commitments [*n* = 1]). One patient finished her participation in the study early due to intolerance of the chamber conditions. Twenty participants completed the study. Ten with T2DM and 10 healthy controls (Table 1).

**Table 1:** Baseline patient characteristics.

	Controls	Type 2 diabetes	Significance
N	10	10	
Gender (male, female)	7/3	3/7	
Age (years)	57.30 ± 10.48	66 ± 7.30	0.07
HbA1c (mmol/mol) (%)	34.20 ± 3.085.3 ± 0.5	41.70 ± 6.386.0 ± 1.0	0.05*
Weight (kg)	73.64 ± 11.57	91.08 ± 15.14	0.01*
BMI (kg/m^2^)	27.34 ± 3.88	31.34 ± 5.17	0.07
Waist/hip ratio	0.87 ± 0.08	0.94 ± 0.09	0.15

*Data are expressed as mean ± SD*.

*BMI, body mass index*.

*Significant difference.

The study was approved by the Humber Bridge Regional Ethics Committee (12/YH/0016) and all participants provided written informed consent. The conduct of the trial was in accordance with ICH GCP and the Declaration of Helsinki.

Participants participated in two visits in the environmental chamber between April 2013 and December 2013. Following the screening visit, participants attended the environmental chamber (Design and Manufacture of Environmental Test Chambers, A3897 – SSR60-20H) on two separate occasions at the University of Hull and were subject to one of two environmental conditions in random order, and then crossed over to the other environmental condition 7 days later. One environmental condition was the control day, when participants were asked to spend 2 h in the environmental chamber at sea level conditions (temperature: 23^o^C, oxygen concentration 21%, humidity 45%). The second environmental condition was when participants were asked to spend 2 h in the environmental chamber in hypoxic conditions, similar to a commercial flight air cabin conditions (temperature: 23^o^C, oxygen concentration 15%, humidity 15%). Baseline and 2 h measurements were taken for each of the measured parameters.

Participants were investigated individually in the chamber and were seated throughout the visits and the same chairs used throughout the study. Participants were asked to refrain from walking during the study and they were allowed to consume water.

Samples for clotting experiments were spun at 3,500 *g* for 15 min immediately after venepuncture, and then frozen at −80°C until batch analysis at the end of the study. Blood was prepared for platelet function and microparticles analysis within 10 min. Participants arrived fasting to eliminate the postprandial effects and were provided lunch after the end of the visits in the environmental chamber.

Fibrin formation and clot structure analysis was undertaken using turbidimetric assays (20). Initial fibrin formation occurs during the “lag” phase, when half-staggered, double-stranded protofibrils are formed (21), and is equivalent to the clotting time. Maximum absorbance is the maximum optical density of the clot, and this is a measure of fibrin network density and fiber thickness. Lysis time is the time from full blood clot formation to 50% lysis that is an indicator of fibrinolytic potential: lysis area is measured as area under the curve. This is a complex measure of clot formation time, clot density, and lysis potential. Higher maximum absorbance, longer lysis time, and larger lysis area are associated with increased cardiovascular risk (22, 23).

Platelets were identified by the pan-platelet marker CD42b from whole blood, and their function was determined by flow cytometry (FACSAria II, BD Biosciences). Samples were labeled with antibodies specifically against the exposed fibrinogen-binding surface receptor to assess activation, and samples were labeled with P-selectin (an antigen of the α-granules) to assess degranulation. These samples were then assessed in the presence of different concentrations of ADP that is an agonist of platelet activation but causes minimal degranulation. Activated platelet inactivation by prostacyclin (PG12) was also undertaken (24).

Endothelial microparticles were characterized by CD106 (with surface VCAM-1 expressed) and CD144 (surface VE-Cadherin expressed) positivity on flow cytometry (BD FACSCalibur). Microparticles were further characterized by their ability to bind annexin V on their surface. Total MPs were calculated using the following formula:

$$\begin{array}{l} \text{Total microparticles(MPs) were derived from }{\text{MPs}}_{\text{annexin V positive}}\\ \;\pm {\text{MPs}}_{\text{annexin V negative}}. \end{array}$$

All antibodies were supplied by BD Biosciences and Thermo Fischer Scientific. AccuCheck counting beads supplied by Thermo Fischer Scientific.

Endothelial function was assessed using PAT arterial tonometry using the EndoPAT 2000 (Itamar Madical Ltd., Israel) that is a reactive hyperemia-based, operator independent device (25, 26). Briefly, 2 disposable, inflatable probes were placed on the index fingers in a sitting position while resting. After 10 min of baseline signal registration, a BP cuff was inflated for 3 min and after release the test was continued for a further 5 min. The EndoPAT 2000 has been validated to assess endothelial function. A RHI lower than a cut-off value of 1.67 has 82% sensitivity and 77% specificity to diagnose coronary endothelial dysfunction (27) and reproducibility is good with an interclass correlation of 0.74.

### Statistical Analysis

Results are expressed as mean ± SD or median (25th, 75th centiles) where applicable. Differences between baseline and corresponding 2-h data were assessed using Student’s paired *t*-test for variables that showed normal distribution, or Wilcoxon signed rank test for variables that violated from the Gaussian distribution. Between group differences were calculated using unpaired *t*-test where the data showed normal distribution, or Mann–Whitney *U*-test where the distribution violated from the Gaussian distribution. Normality testing was performed using the Shapiro–Wilk test. Correlation was calculated using Pearson’s correlation test. Statistical analysis was performed using SPSS 19.0.0 (IBM Corp., New York, NY, USA) and statistical significance was defined as (*p* < 0.05). As this was a pilot study, no power analysis was applicable; however, power and sample size for pilot studies have been reviewed by Birkett and Day (28) who concluded that a minimum of 20 degrees-of-freedom was required to estimate effect size and variability, hence, we recruited 10 participants per group.

## Results

Patient baseline characteristics are shown in Table 1 with participants with T2DM having a mean disease duration of 5.75 ± 3.58 years. There was a significant difference in weight between healthy controls and patients with diabetes; however, BMI did not differ significantly.

Maximal absorption indicating clot density was increased in both normal and participants with T2DM (*p* = 0.04, *p* < 0.01) following the SF (Table 2), but did not differ on the control day. There was no difference in baseline clotting parameters between healthy controls and participants with T2DM (data not shown). Lag time, that is equivalent to the clotting time, did not differ following the SF (Table 2), while it significantly decreased during the control day. Lysis time and lysis area did not differ at baseline between groups, on the control day and following the SF (Table 2).

**Table 2:** Clotting indices in healthy volunteers and participants with type 2 diabetes (T2DM) before and after simulated flight (SF) and exposed to oxygen concentration 15% and humidity 15%.

	Hypoxia							
	Controls				T2DM			
	Before	After	*p*	CI/z (N)	Before	After	*p*	CI/z (N)
Lag time	455.00 ± 52.99	424.40 ± 63.02	0.17	−16.35 to 77.55	446.90 ± 93.57	450.50 ± 61.02	0.89	−58.39 to 51.19
Maximum absorption	0.375 ± 0.050	0.390 ± 0.053	0.04*	−0.32 to 0.01	0.378 ± 0.076	0.397 ± 0.089	0.01*	−0.03 to 0.01
Lysis time	1500.33 (1261.25, 1822.92)	1570.83 (1386.92, 1868.00)	0.17	–1.38 (4)	1422.33 (1330.00, 1458.42)	1362.67 (1276.17, 1507.42)	0.29	−1.07 (7)
Lysis area	658.20 ± 289.27	675.11 ± 215.61	0.64	−95.23 to 61.40	556.05 ± 157.71	591.34 ± 219.85	0.29	−106.53 to 35.94

*Maximal absorption indicating clot density was increased in both normal and T2DM participants, though other functional clot elements were unchanged following the SF*.

*Significant difference.

There was an increase in platelet activation for both fibrinogen and p-selectin following the SF though platelet response to ADP (Table 3) and sensitivity to PGI2 was unchanged (Table 4). There was no difference in baseline platelet function between controls and participants with T2DM nor on the control day (data not shown).

**Table 3:** Platelet activation and response to ADP prior to and after the relative hypoxia of the simulated flight (SF) intervention.

	Fibrinogen binding							
	Controls				Type 2 diabetes (T2DM)			
	Before	After	*p*	CI	Before	After	*p*	CI
Basal	2.03 ± 0.50	2.39 ± 1.12	0.30	−1.10 to 0.38	1.56 ± 0.45	2.17 ± 0.50	0.05*	−1.21 to 0.01
ADP 0.1 µm	5.04 ± 2.63	4.70 ± 2.08	0.62	−1.18 to 1.86	5.23 ± 3.27	5.42 ± 2.83	0.90	−3.32 to 2.95
ADP 1 µm	45.50 ± 16.42	46.62 ± 11.92	0.63	−6.14 to 3.91	49.14 ± 14.39	47.28 ± 13.26	0.79	−14.07 to 17.81
ADP 10 µm	63.17 ± 12.89	67.52 ± 10.25	0.09	−9.52 to 0.83	68.84 ± 14.19	55.03 ± 9.86	0.60	−11.21 to 18.09

	**P-selectin expression**							
	**Controls**				**T2DM**			
	**Before**	**After**	***p***	**CI/z (N)**	**Before**	**After**	***p***	**CI/z (N)**

Basal	2.18 (1.65, 5.58)	2.08 (1.31, 5.60)	0.50	−0.59 (5)	2.35 (1.80, 6.55)	6.20 (3.28, 7.08)	0.05*	−1.96 (2)
ADP 0.1 µm	6.61 ± 3.97	5.60 ± 3.28	0.40	−1.56 to 3.58	8.41 ± 4.56	9.53 ± 3.60	0.56	−5.42 to 3.17
ADP 1 µm	48.43 ± 19.61	47.99 ± 15.62	0.91	−7.80 to 8.67	54.57 ± 14.14	55.03 ± 9.86	0.94	−14.69 to 13.77
ADP 10 µm	67.58 ± 18.04	70.68 ± 13.60	0.26	−8.96 to 2.75	73.89 ± 12.20	77.42 ± 9.26	0.52	−15.73 to 8.68

*Base line platelet activation following the SF was seen for participants with T2DM that was not exacerbated by ADP stimulated platelet activation. This suggests that whilst the diabetes platelet may be more prone to being activated by the SF, the activated response does not differ to normal*.

*Significant difference.

**Table 4:** Platelet sensitivity to PGI_2_ prior to and after the relative hypoxia of the simulated flight (SF) intervention showing no difference in deactivation of the ADP activated platelets between controls and patients with type 2 diabetes (T2DM).

	Fibrinogen binding							
	Controls				T2DM			
	Before	After	*p*	CI/z (N)	Before	After	*p*	CI/z (N)
PGI_2_ 1 nM ± ADP	42.40 ± 15.58	37.37 ± 17.85	0.08	−0.72 to 10.77	36.22 ± 8.83	35.07 ± 19.28	0.89	−18.08 to 20.38
PGI_2_ 10 nM ± ADP	56.72 ± 15.73	54.62 ± 14.08	0.55	−5.57 to 9.78	51.40 ± 11.06	49.10 ± 19.36	0.78	−16.39 to 20.97
PGI_2_ 100 nM ± ADP	95.98 (86.68, 97.09)	94.19 (91.06, 95.62)	0.88	−0.15 (6)	92.41 (83.82, 96.49)	95.45 (93.69, 96.25)	0.26	−1.13 (3)

	**P-selectin expression**							
	**Controls**				**T2DM**			
	**Before**	**After**	***p***	**CI/z (N)**	**Before**	**After**	***p***	**CI/z (N)**

PGI_2_ 1 nM ± ADP	41.71 ± 15.98	40.25 ± 18.35	0.54	−3.66 to 6.57	33.88 ± 9.15	36.42 ± 7.97	0.55	−11.97 to 6.89
PGI_2_ 10 nM ± ADP	50.90 (49.31, 57.06)	57.85 (39.63, 70.93)	0.96	−0.05 (4)	49.52 (33.42, 53.04)	53.73 (40.88, 55.12)	0.59	−0.53 (4)
PGI_2_ 100 nM ± ADP	91.99 (84.57, 94.79)	92.82 (86.96, 95.84)	0.66	−0.96 (6)	86.53 (78.21, 91.43)	89.15 (84.64, 92.57)	0.59	−0.53 (4)

Annexin V negative and total VCAM-1 positive MPs were significantly higher in participants with T2DM compared to controls (248 ± 130 vs. 144 ± 68, *p* = 0.04; 314 ± 130 vs. 196 ± 65, *p* = 0.02, respectively) indicating impaired baseline endothelial function in participants with T2DM compared to the controls. However, there was no increase in MP counts during the SF, and the RHI also remained unchanged following the SF. Neither of these parameters changed during the control experiment (data not shown).

Results from the control day are presented in Tables 5 and 6.

**Table 5:** Results from the control day.

	Normal day							
	Controls				Type 2 diabetes (T2DM)			
	Before	After	*p*	CI/z (N)	Before	After	*p*	CI/z (N)
Lag time	436.11 ± 31.19	406.11 ± 45.50	0.04*	−95.23 to 61.41	466.50 ± 76.64	422.70 ± 78.36	0.02*	9.28 to 78.32
Maximum absorption	0.404 ± 0.068	0.413 ± 0.064	0.12	1.20 to 58.80	0.340 ± 0.082	0.374 ± 0.109	0.31	−0.03 to 0.08
Lysis time	1628.33 (1284.83, 2100.50)	1632.33 (1305.25, 2488.25)	0.11	−1.60 (2)	1432.33 (1325.25, 1578.67)	1427.83 (1393.50, 1733.00)	0.20	−1.27 (4)
Lysis area	732.77 (434.31, 1250.34)	772.05 (427.26, 1227.70)	0.09	−1.72 (1)	607.38 (496.64, 683.83)	635.65 (537.14, 680.33)	0.33	−0.97 (3)

*Clotting indices in healthy volunteers and participants with T2DM during the control experiment. There was decrease in lag time in both groups*.

**Table 6:** Results from the control day.

	Fibrinogen binding							
	Controls				T2DM			
	Before	After	*p*	CI/z (N)	Before	After	*p*	CI/z (N)
Basal	2.38 (1.89, 2.86)	2.08 (1.85, 2.33)	0.24	−1.17 (7)	2.48 (2.34, 3.08)	2.25 (2.08, 2.66)	0.61	−0.51 (5)
ADP 0.1 µm	4.55 (3.63, 7.00)	3.90 (3.01, 5.86)	0.51	−0.66 (6)	7.30 (2.74, 12.15)	5.50 (3.41, 8.26)	0.14	−1.48 (6)
ADP 1 µm	42.92 ± 9.96	44.99 ± 11.32	0.32	−6.49 to 2.35	48.49 ± 18.10	45.70 ± 18.13	0.14	−1.06 to 6.64
ADP 10 µm	64.12 ± 12.80	63.13 ± 11.16	0.65	−3.80 to 5.77	66.53 ± 17.89	64.61 ± 17.26	0.39	−2.86 to 6.70

	**P-selectin expression**							
	**Controls**				**T2DM**			
	**Before**	**After**	***p***	**CI/z (N)**	**Before**	**After**	***p***	**CI/z (N)**

Basal	1.70 (1.54, 4.03)	1.60 (1.53, 2.54)	0.21	−1.25 (6)	2.10 (1.70, 2.43)	1.85 (1.60, 2.18)	0.48	−0.71 (6)
ADP 0.1 µm	4.20 (2.94, 6.71)	3.43 (2.90, 6.20)	0.96	−0.05 (5)	4.98 (3.25, 10.06)	4.48 (3.75, 7.15)	0.39	−0.87 (5)
ADP 1 µm	40.37 ± 11.94	43.86 ± 11.69	0.12	−8.14 to 1.16	42.92 ± 17.36	41.81 ± 14.72	0.52	−2.63 to 4.85
ADP 10 µm	62.93 ± 14.73	63.71 ± 12.46	0.75	−6.05 to 4.50	62.59 ± 18.08	62.95 ± 14.48	0.85	−4.60 to 3.89

	**Fibrinogen binding**							
	**Controls**				**T2DM**			
	**Before**	**After**	***p***	**CI/z (N)**	**Before**	**After**	***p***	**CI/z (N)**

PGI_2_ 1 nM ± ADP	44.00 ± 11.38	40.33 ± 11.07	0.28	−3.57 to 10.91	40.03 ± 15.47	43.96 ± 19.73	0.51	−16.89 to 9.02
PGI_2_ 10 nM ± ADP	58.27 ± 16.65	57.55 ± 12.37	0.92	−15.07 to 16.50	50.51 ± 18.68	53.98 ± 17.50	0.37	−11.83 to 4.89
PGI_2_ 100 nM ± ADP	93.69 (83.17, 96.36)	94.50 (87.02, 96.86)	0.96	−0.05 (5)	93.61 (82.93, 95.20)	91.75 (84.79, 95.38)	0.39	−0.87 (3)

	**P-selectin expression**							
	**Controls**				**T2DM**			
	**Before**	**After**	***p***	**CI/z (N)**	**Before**	**After**	***p***	**CI/z (N)**

PGI_2_ 1 nM ± ADP	44.98 ± 8.97	42.35 ± 10.84	0.43	4.58 to 9.48	43.47 ± 14.91	43.96 ± 19.73	0.51	−17.82 to 9.46
PGI_2_ 10 nM ± ADP	60.35 ± 16.21	58.22 ± 14.62	0.78	−14.26 to 18.51	54.02 ± 16.46	56.70 ± 13.23	0.38	−9.28 to 3.92
PGI_2_ 100 nM ± ADP	93.98 (83.17, 96.36)	92.93 (85.83, 96.95)	0.96	−0.05 (5)	93.91 (85.80, 95.27)	91.86 (87.79, 96.00)	0.58	−0.56 (4)

*Platelet function in healthy volunteers and patients with T2DM during the control experiment*.

## Discussion

This is the first study to assess the physiological changes that might affect thrombus formation when simulating a short duration commercial air flight, comparing participants with T2DM and healthy controls. Short-haul travel is very common and has not been investigated, and specific studies exploring the risk for patients with diabetes to develop travel-related VTE are lacking. Given that hypobaric hypoxia showed prothrombotic changes at 2 h into exposure (9), this was the rationale for choosing the 2 h time frame.

Data suggest increased risk to develop fatal pulmonary embolism even after a 3-h duration of air travel (8). A recent case report describes bilateral pulmonary embolism after a short-haul flight in a man with multiple risk factors (29). In a hypobaric hypoxia study involving 20 healthy male volunteers, the authors showed that prothrombin 1 and 2 fragment levels reached maximum levels at 2 h into exposure (9). These data justify the short duration of our study visits in the absence of relevant data involving patients with T2DM.

There was increased maximum clot absorption following the SF indicating denser clot structure for both normal and participants with T2DM. This suggests that some elements of the clotting mechanism with greater clot density may be affected both in healthy participants and participants with T2DM equally. This suggests that should a thrombus form, its dissolution may be more difficult and prolonged (13). Conversely the lag (clotting time) and the lysis time here did not differ between normal and diabetes before or after the SF suggesting that enhanced clot formation would seem unlikely to occur and is reassuring that is not an enhanced risk for VTE. Lag time decreased in both groups during the control day, which may represent a circadian rhythm (30). To account for any circadian changes, participants entered and exited the environmental chamber at the same time during the different visits. This is superficially discrepant to a report detailing that there were no differences in soluble coagulation factors following a hypoxic SF, but the detailed clotting parameters in that study were not performed (31).

Fibrinogen binding and P-selectin expression on platelets were increased in T2DM participants, but not controls, after the hypoxia of the SF indicating platelet hyperactivity. However, the incremental degree of platelet activation with ADP was not enhanced and did not differ between normal and participants with T2DM. Furthermore, inactivation of the ADP activated platelets by PGI2 was not altered between groups or after the SF. This suggests that the SF increased and perhaps would prime platelet activation, but once activated then the response would not differ to normal, and platelet inactivation mechanisms were also unaffected. These findings are in accord with data showing that T2DM is associated with altered platelet function and metabolism (32). These data differ to the report of plasma soluble p-selectin and beta-thromboglobulin being unchanged following 8 h SF; however, those serum markers are relatively insensitive to platelet changes (31). These data suggest that whilst basal platelet function may be affected by diabetes that there was no enhanced functional changes due to aircraft environmental conditions as shown by ADP or PGI2 being unaffected.

There was an increase in baseline endothelial MPs in T2DM in comparison to control that was expected as a measure of EC dysfunction (33), but MPs and the RHI were unaffected by the SF indicating no further deterioration of endothelial function.

Interestingly, there was a significant decrease in lag time in both healthy controls and patient with T2DM during the control day while there was no change during the hypoxic experiment and this may related to circadian changes (30). When comparing the% changes during the normoxic and hypoxic experiments, there was no significant difference. These with our other data are in line with a previously published cross-over study where increase in the thrombin–antithrombin complex was reported following an 8-h flight compared to an 8-h movie marathon or 8 h of normal daily activity where greater changes were observed in participants who had Factor V Leiden mutation or were taking OCPs (34). In a different study, 20 participants of whom 10 had risk factors to develop VTE took part in a long-haul flight. There was increased activity of FVII and FVIII with suppressed fibrinolysis though there was no difference between the two groups (35).

A meta-analysis reported that there was a 1.4-fold increased risk to develop VTE in participants with diabetes (36). However, the data reported here are in accord with others suggesting that a SF would not cause an elevated VTE risk in T2DM (31).

The main limitation of this study is the small number of participants for both those with T2DM and the healthy controls. The random order of the environmental exposure circumvented any bias in the results that may have been due to only one exposure followed by the other. The cross over design was preferred as each participant could as their own control, particularly as it was unclear if normoxia (control) would have specific individual effects by itself. Gender distribution between the groups may have influenced the results; however, given the limited differences seen between the normal controls and T2DM in this study, it would suggest that short haul commercial flying poses no additional hazards for those with T2DM.

In conclusion, there was increased clot density following a hypoxic SF, but dynamic tests of clotting, platelet function, and endothelial function did not differ suggesting that short haul commercial flying adds no additional hazard for VTE in T2DM compared to normal healthy control participants.

## Ethics Statement

The study was approved by the Humber Bridge Regional Ethics Committee (12/YH/0016) and all participants provided written informed consent. The conduct of the trial was in accordance with ICH GCP and the Declaration of Helsinki.

## Author Contributions

JK performed the statistical analysis, researched data, and wrote the paper. BS performed and interpreted the platelet function tests. AQ, TS, RA, LM, KM, AG, and EK reviewed and edited the manuscript. SA reviewed and edited the manuscript and is the guarantor of the manuscript. All authors approved the final submission of the manuscript.

## Conflict of Interest Statement

The authors declare that the research was conducted in the absence of any commercial or financial relationships that could be construed as a potential conflict of interest.
