# Corrigendum: The Effect of a Simulated Commercial Flight Environment With Hypoxia and Low Humidity on Clotting, Platelet, and Endothelial Function in Participants With Type 2 Diabetes – A Cross-Over Study

**DOI:** 10.3389/fendo.2018.00301

**Published:** 2018-06-07

**Authors:** Judit Konya, Benjamin E. J. Spurgeon, Ahmed Al Qaissi, Thozhukat Sathyapalan, Ramzi Ajjan, Leigh Madden, Khalid M. Naseem, Andrew Thomas Garrett, Eric Kilpatrick, Stephen L. Atkin

**Affiliations:** ^1^Department of Academic Endocrinology, Diabetes and Metabolism, Hull York Medical School, Hull, United Kingdom; ^2^School of Medicine, Leeds Institute for Cardiovascular and Metabolic Medicine, University of Leeds, Leeds, United Kingdom; ^3^Hull York Medical School, University of Hull, Hull, United Kingdom; ^4^Department of Sport, Health and Exercise Science, University of Hull, Hull, United Kingdom; ^5^Sidra Medical Research Centre, Doha, Qatar; ^6^Department of Medicine, Weill Cornell Medicine Qatar, Doha, Qatar

**Keywords:** type 2 diabetes, hypoxia, flight simulation, platelet function, endothelial function, clotting

Benjamin E. J. Spurgeon was not included as an author in the published article. The updated Author Contributions statement appears below.

The authors apologize for this error and state that this does not change the scientific conclusions of the article in any way.

The original article has been updated.

## Author Contributions

JK performed the statistical analysis, researched data, and wrote the paper. BS performed and interpreted the platelet function tests. AQ, TS, RA, LM, KM, AG, and EK reviewed and edited the manuscript. SA reviewed and edited the manuscript and is the guarantor of the manuscript. All authors approved the final submission of the manuscript.

## Conflict of Interest Statement

The authors declare that the research was conducted in the absence of any commercial or financial relationships that could be construed as a potential conflict of interest.

